# Donor–acceptor graphene-based hybrid materials facilitating photo-induced electron-transfer reactions

**DOI:** 10.3762/bjnano.5.170

**Published:** 2014-09-18

**Authors:** Anastasios Stergiou, Georgia Pagona, Nikos Tagmatarchis

**Affiliations:** 1 Theoretical and Physical Chemistry Institute, National Hellenic Research Foundation, 48 Vassileos Constantinou Avenue, 11635 Athens, Greece

**Keywords:** donor–acceptor, electron-transfer, functionalization, graphene, photophysical properties

## Abstract

Graphene research and in particular the topic of chemical functionalization of graphene has exploded in the last decade. The main aim is to increase the solubility and thereby enhance the processability of the material, which is otherwise insoluble and inapplicable for technological applications when stacked in the form of graphite. To this end, initially, graphite was oxidized under harsh conditions to yield exfoliated graphene oxide sheets that are soluble in aqueous media and amenable to chemical modifications due to the presence of carboxylic acid groups at the edges of the lattice. However, it was obvious that the high-defect framework of graphene oxide cannot be readily utilized in applications that are governed by charge-transfer processes, for example, in solar cells. Alternatively, exfoliated graphene has been applied toward the realization of some donor–acceptor hybrid materials with photo- and/or electro-active components. The main body of research regarding obtaining donor–acceptor hybrid materials based on graphene to facilitate charge-transfer phenomena, which is reviewed here, concerns the incorporation of porphyrins and phthalocyanines onto graphene sheets. Through illustrative schemes, the preparation and most importantly the photophysical properties of such graphene-based ensembles will be described. Important parameters, such as the generation of the charge-separated state upon photoexcitation of the organic electron donor, the lifetimes of the charge-separation and charge-recombination as well as the incident-photon-to-current efficiency value for some donor–acceptor graphene-based hybrids, will be discussed.

## Introduction

Among the outstanding forms of carbon nanostructures, graphene, a single layer of carbon, is a newly available material exhibiting unique mechanical [1] and electronic properties [2] and can be described as one of the most extensively examined materials of recent years [3–4]. Diverse approaches have been developed to obtain graphene sheets, including the mechanical cleavage of graphite [3], chemical vapor deposition on metal surfaces [5–6], liquid exfoliation via sonication [7–8], dissolution in superacids such as chlorosulfonic acid [9] and ball milling [10]. However, a major drawback of graphene, likewise of carbon nanotubes, stems from its insolubility in all solvents, which impedes the chemical manipulation toward applications. There are two main routes to overcome this hurdle. Namely, this can be accomplished by starting with water-soluble graphene oxide (GO), which can be reduced to the so-called reduced graphene oxide (rGO), followed by post-modification to acquire functionalized graphene [11]. However, the reduction of GO sometimes leads to amorphous carbon [12], and often the graphene sp^2^-network is incompletely restored. Therefore the properties of the resulting rGO significantly deviate from those of pristine graphene. Hence, this particular approach in not suitable for applications in which the novel electronic properties of graphene are of primary importance. Alternatively, wet exfoliation of graphite followed by functionalization is a more efficient strategy. Although functionalization of exfoliated graphene can be achieved by either covalent anchoring of organic molecules onto the graphene lattice [13] or supramolecularly, by π–π stacking and/or van der Waals interactions [14], the latter methodology suffers from weak interactions between the two species (i.e., graphene and organic units), which often leads to a loss of the organic moiety. Apparently, covalent functionalization of exfoliated graphene, in which the organic unit is tightly attached on the graphene network, is the method of choice for preparing novel donor–acceptor hybrid materials that can potentially facilitate photo-induced electron-transfer phenomena.

Single-layer, bilayer and oligo-layer graphene sheets have been utilized to fabricate donor–acceptor ensembles potentially useful in energy conversion schemes. Actually, electrons can travel without scattering around the 2D crystal structure of graphene [15], thus guaranteeing a continuous lossless electron flow. In addition, considering that carbon nanotubes are generally produced as a mixture of metallic and semiconducting components, and also sometimes retain significant amounts of metal nanoparticles as impurities, the advantageous character of graphene in the construction of donor–acceptor systems is easily understood. Although the field is in its infancy, a few hybrid materials composed of graphene and photoactive components, such as porphyrins and phthalocyanines, have been prepared and evaluated regarding photo-induced charge transfer phenomena [16–18]. Moreover, semiconducting quantum dots such as CdS [19–22], CdSe [23–26], CdTe [27–28], ZnO [29–30] and composites thereof, for instance, Au nanoparticles [31–32] have been incorporated into graphene sheets (GO or rGO) to yield donor–acceptor systems. However, the aim of this mini-review is to highlight recent advances in the preparation of graphene-based hybrid materials with organic electron donors for energy conversion. Additonally, routes and strategies for the chemical functionalization of graphene with photoactive electron donors will be presented and common techniques for characterizing and evaluating the photophysical properties of the materials will be discussed.

## Review

### Chemical functionalization toward the formation of donor–acceptor hybrids

**Scheme 1:**
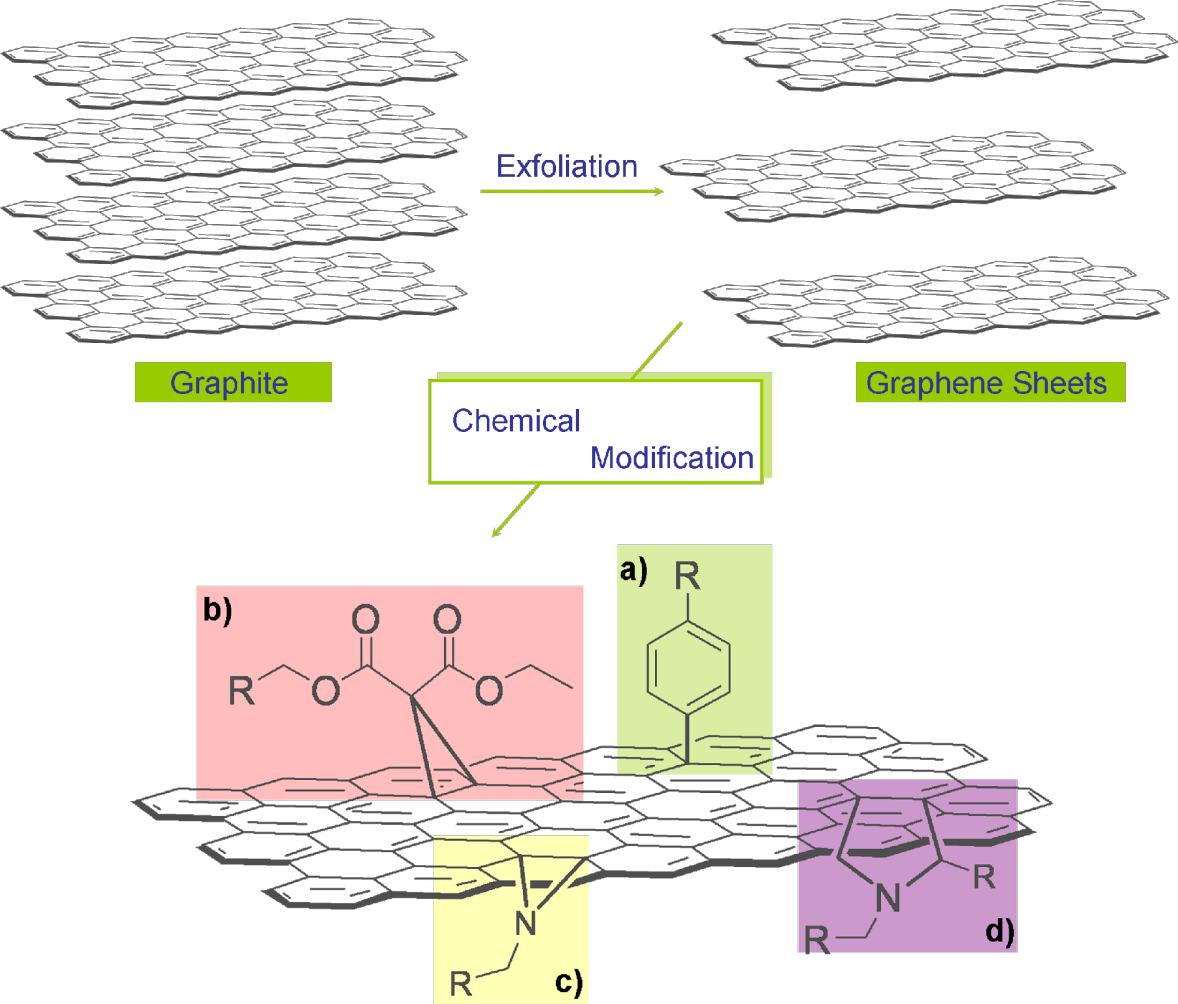
General chemical modification routes for exfoliated graphene sheets. (a) [3 + 2] 1,3-dipolar cycloaddition of in situ generated azomethine ylides, (b) [1 + 2] Bingel cycloaddition, (c) aryl diazonium addition, and (d) azide addition.

A plethora of soluble and easy-to-handle graphene-based materials have been prepared and methodologies for the functionalization of graphene have already been reviewed [12,33–37]. In Scheme 1, a collection of the major chemical reactions leading to the covalent modification of a graphene framework are displayed. Briefly, after the exfoliation of graphite, the following reactions can be performed to modify the graphene sheet (summarized in Scheme 1):

1. [3 + 2] 1,3-Dipolar cycloaddition of in situ generated azomethine ylides to introduce fused pyrrolidine rings into the skeleton of graphene [38]. Azomethine ylides are organic 1,3-dipoles possessing a carbanion next to an iminium ion and can be readily produced upon decarboxylation of the iminium salts derived from the condensation of α-amino acids with aldehydes or ketones. When azomethine ylides are added to graphene, a fused pyrrolidine ring is formed at the junction between two six-membered rings of the graphene lattice. Notably, functionalized aldehydes lead to the formation of 2-substituted pyrrolidines, whereas the reaction with N-substituted glycines leads to N-substituted pyrrolidines, thus allowing for the preparation of numerous custom-synthesized graphene-based materials;
2. [1 + 2] Cycloaddition of malonate derivatives, the so-called Bingel reaction, yielding cyclopropane rings on graphene [39]. This versatile modification involves the generation of carbon nucleophiles from α-halo esters and their subsequent regioselective addition to graphene. In general, the addition takes place on double bonds between two six-membered rings present on graphene, yielding methano-modified graphene-based materials. Moreover, modifications of the original Bingel reaction exist, utilizing (i) carbanionic precursors to methano-modified graphene other than malonates, and (ii) alternative pathways generating the reactive monohalomalonate intermediate in situ;
3. Addition of in situ generated aryl diazonium salts, proceeding via the release of dinitrogen [40]. The reaction mechanism involves an electron transfer from graphene to the diazonium salt, resulting in the formation of a radical aryl unit, which subsequently adds to the sp^2^-carbon lattice of graphene;
4. Addition of azides forming aziridine adducts onto graphene [41–43]. The particular functionalization proceeds via nitrenes as generated upon the thermal (or photochemical) decomposition of azides and the liberation of dinitrogen.

### Characterization, charge-separation and incident-photon-to-current efficiency

Raman spectroscopy is an extremely useful tool for characterizing graphene-based materials. Pristine graphite exhibits two characteristic Raman modes, namely the G-band, due to the presence of sp^2^-hybridized carbon atoms at 1585 cm^−1^ and the 2D band at a higher frequency of around 2725 cm^−1^. In addition, functionalized graphene sheets exhibit a new band, the so-called disorder D-band at around 1350 cm^−1^, due to the presence of sp^3^-hybridized carbon atoms, present at defects and/or anchor sites of functional groups. Furthermore, the 2D band in functionalized graphene shifts to lower frequencies and narrows compared to that of graphite. Thermal gravimetric analysis gives information about the degree of functionalization in modified graphene sheets because the covalent attachment of the organic moieties can be thermally eliminated, while intact graphite is thermally stable under inert atmosphere. Moreover, microscopy techniques such as TEM and AFM offer significant insight into the morphology of graphene and especially the number of layers in a particular sample. Overall, based on such complementary spectroscopic, microscopy and thermal techniques graphene-based hybrid materials can be qualitatively monitored, while at the same time information about the dimensions and purity is obtained.

**Figure 1:**
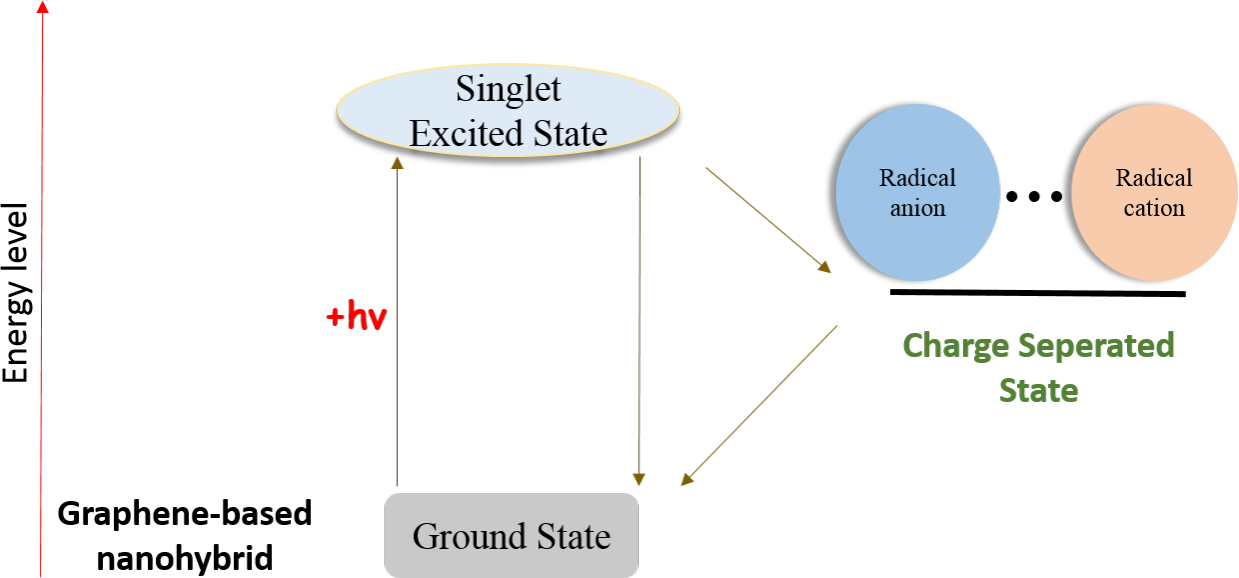
Illustrative energy diagram for the photo-induced formation of the charge-separated state of graphene-based hybrid materials.

With regard to energy conversion schemes, the challenge for constructing robust and functional architectures made of graphene and photo- and/or electro-active components is met by considering and applying to a large extent the aforementioned functionalization strategies. In this respect, a few covalently linked graphene sheets with organic electron-donor moieties to facilitate photo-induced electron-transfer processes have been synthesized. Briefly, photo-induced excitation of the organic electron donor in the graphene-based hybrid materials results on the formation of the singlet-excited state of the organic component. Then, charge-separation takes place and the efficiency of the whole process is governed by how fast or slow the recombination of charges occurs. A schematic description of such a process, which sometimes may be quite complex involving triplet states as derived upon intersystem crossing, is presented in Figure 1.

Usually, the photoelectrochemical measurements of graphene-based hybrid materials with photoactive organic electron donors are carried out in a three-compartment cell using a potentiostat, employing a saturated calomel reference electrode, a working electrode and a Pt wire gauze counter electrode. This configuration allows for photocurrent measurements under electrochemical bias. Electrophoretic deposition is applied to fabricate films of the graphene-based hybrid material onto an optically transparent electrode (OTE) covered with nanostructured SnO_2_. Typically, a suspension of the graphene-based hybrid material in THF is transferred to a cuvette, then, two OTEs cut from conducting glass are inserted, and a DC electric field is applied. Then, the graphene-based hybrid material from the suspension is driven to the surface of the positive electrode, and a robust thin-film (abbreviated as OTE/SnO_2_/graphene-based hybrid material) is deposited within a short period of time. The photocurrent action spectrum of the OTE/SnO_2_/graphene-based hybrid material electrode is evaluated by examining the wavelength dependence of the incident-photon-to-current conversion efficiency (IPCE). The IPCE values are calculated by normalizing the photocurrent densities by energy and intensity of the incident light according to the following equation:


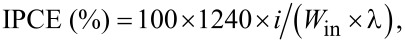


where *i* is the photocurrent density (A·cm^−2^), *W*_in_ is the incident light intensity (W·cm^−2^), and λ is the excitation wavelength (nm).

### Donor–acceptor hybrids based on graphene oxide or reduced graphene oxide

**Figure 2:**
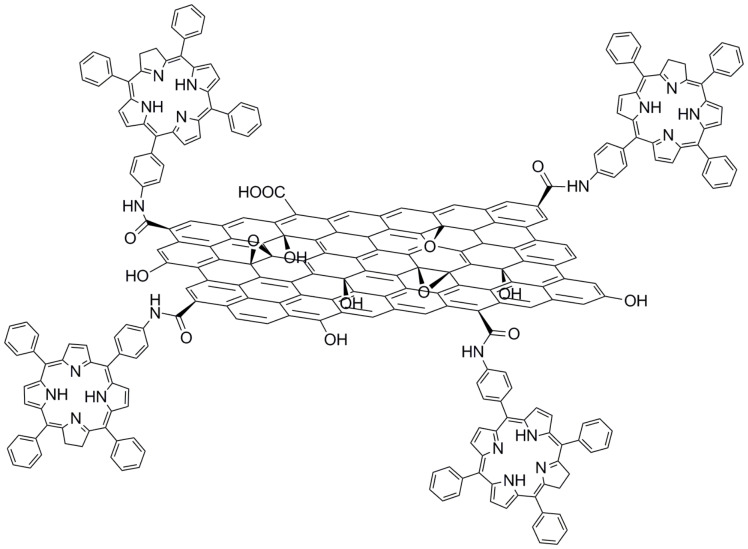
Tetraphenylporphyrin (TTP) condensed onto graphene oxide (GO) yielding GO–TPP hybrid material [44].

The unique and notable features of porphyrins and phthalocyanines as light-harvesting antennas with high extinction coefficients and remarkable redox properties make them promising electron donors to be associated with graphene. The very first approach of associating porphyrins with graphene was made by condensing an amino-modified tetraphenylporphyrin (TPP) to the carboxylic functionalities present in GO [44] providing the GO–TPP hybrid structure shown in Figure 2. The observed luminescence quenching of TPP in the GO–TPP hybrid material is indicative of a strong electronic interaction between TPP in the excited state and GO. Possible pathways for the fluorescence quenching of TPP are attributed either to an electron transfer or to an energy transfer to GO. Additionally, the formation of the charge-separated state (GO)^•−^–(TPP)^•+^ was shown to have an energy gap of 0.87 eV, while the IPCE was calculated to be 1.3% [45].

Along the same lines, when hydroxy-functionalized porphyrin (H_2_P) was condensed onto GO sheets, the GO–H_2_P hybrid material showed enhanced non-linear optical (NLO) properties, which were mainly ascribed to photo-induced electron-transfer from H_2_P to GO [46]. Similarly, an amino-modified zinc phthalocyanine (ZnPc) was conjugated to GO furnishing the GO–ZnPc hybrid, which showed enhanced NLO properties due to strong electronic interactions between the donor and acceptor components within the nanohybrid material [47].

As a different route taken to associate porphyrin units with GO, an imidazolium ionic-liquid was conjugated to GO forming an amide bond. The presence of the positively charged imidazolium moiety allows the modulation of the dispersibility of the modified GO material in aqueous media and organic solvents by simply exchanging and altering the counter anion of the imidazolium unit. More importantly, when an anionic porphyrin was incorporated as counter anion to the GO–ionic-liquid hybrid material, electronic interactions between the porphyrin and the GO lattice were identified [48].

Zinc(II) phthalocyanine (ZnPc) as electron donor and C_60_ were added to GO through an esterification reaction between the carboxyl groups of GO and the hydroxy groups present on ZnPc as well as on the fullerene derivative. Photoexcitation of ZnPc–GO–C_60_ at 390 nm, the wavelength at which ZnPc was predominantly excited, resulted in the identification of the first singlet excited state of ZnPc. However, photoexcitation at 532 nm, at which mainly C_60_ was excited, revealed the formation of both a ZnPc radical cation and a C_60_ radical anion. The latter results, as obtained from nanosecond transient absorption spectroscopy measurements, ascertain the formation of a charge-separated state in the ZnPc–GO–C_60_ hybrid material [49].

**Figure 3:**
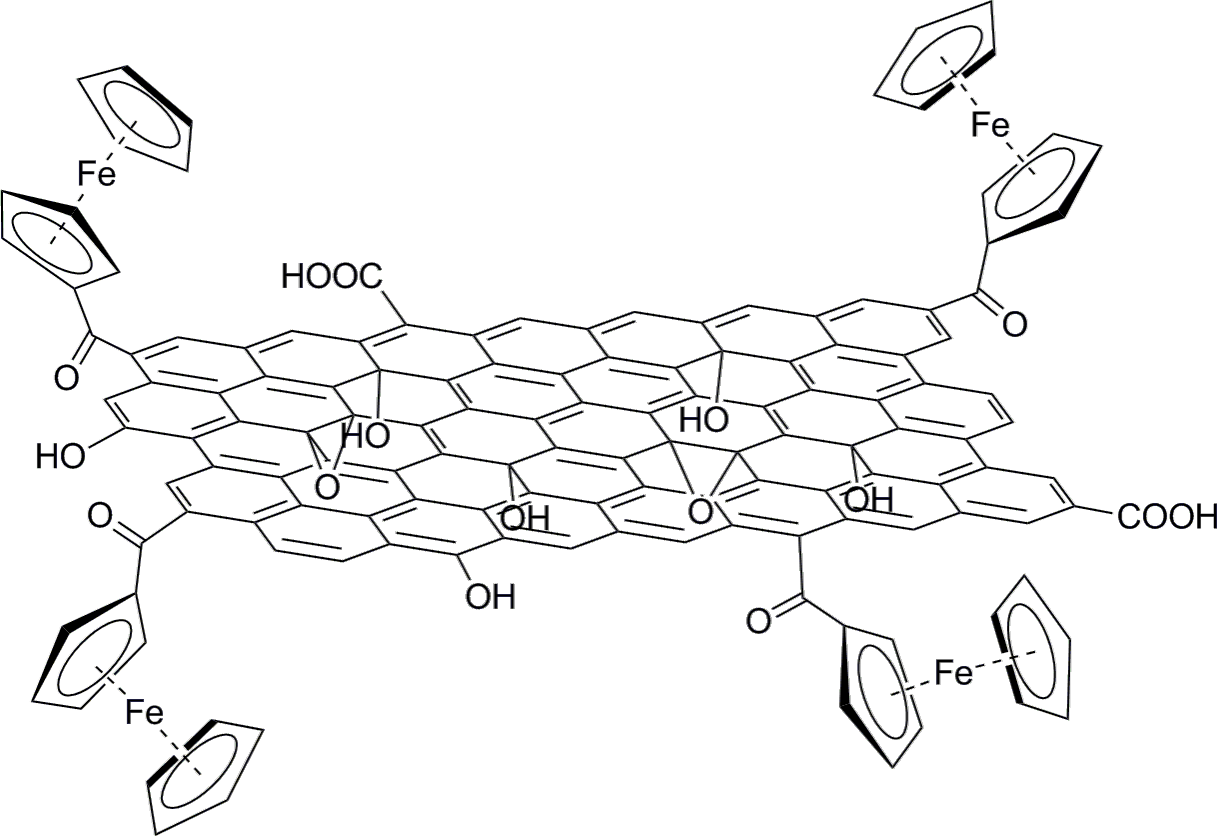
Ferrocene units anchored to graphene oxide (GO) forming a GO–Fc hybrid material [50].

Recently, the fabrication of a composite of GO and ferrocene moieties (GO–Fc, see Figure 3) was reported [50]. Although, the study refers to graphene oxide, it is the first report that describes the combination of graphene and ferrocene properties through chemical bonding. The photo-responsiveness of the developed composite was investigated by fabricating a Au/GO–Fc/Au device. Interestingly, a significant enhancement in current density under illumination with light was observed, which suggests charge-transfer processes within the GO–Fc hybrid material.

**Scheme 2:**
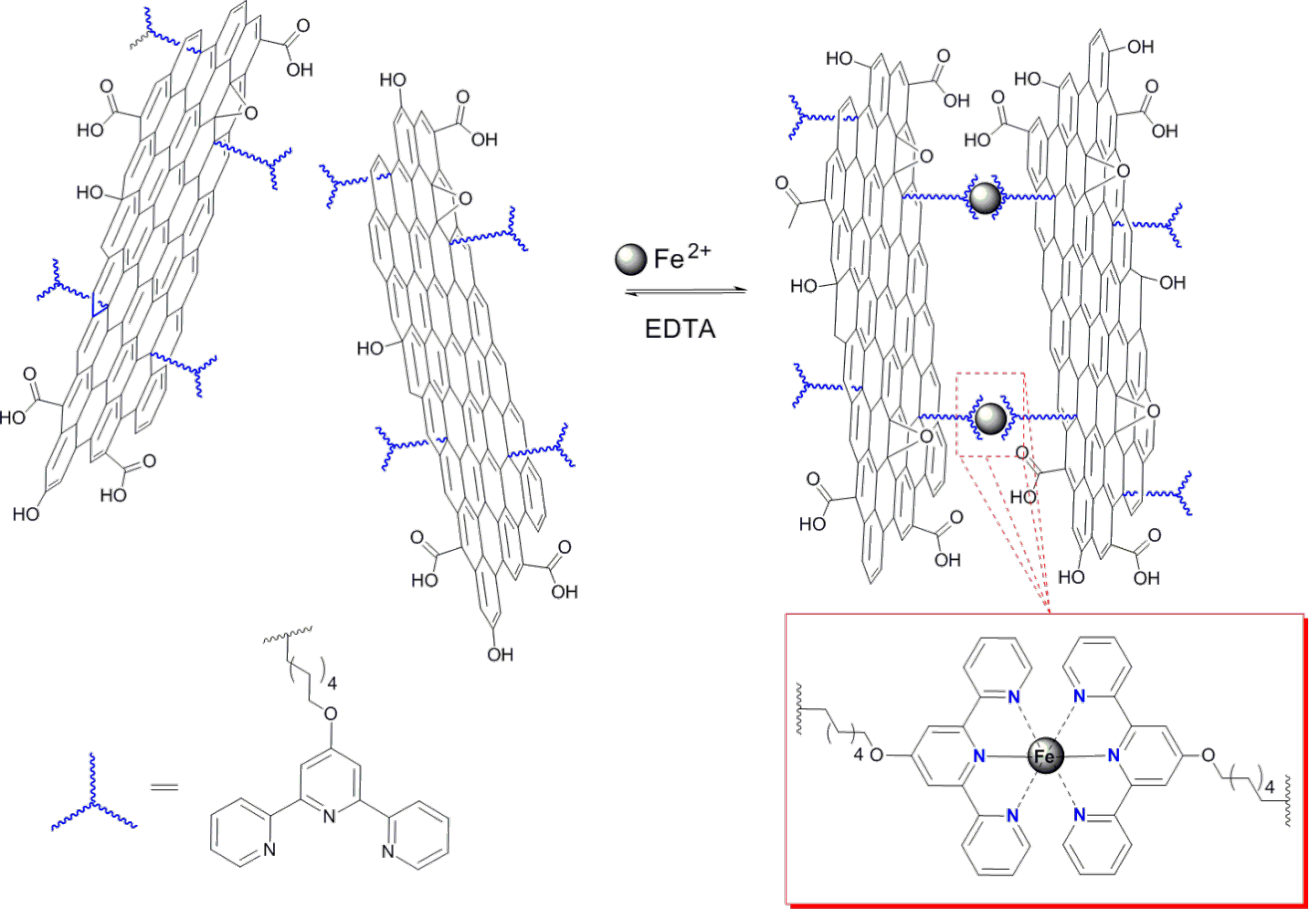
Iron(II) coordinated on terpyridine (tpy) moieties covalently anchored to graphene oxide (GO) forming GO–tpy–Fe hybrids [51].

As described above, cations of transition metals are often present in chromophores (the central metal core of Pcs, porphyrins etc.) and take part in the electron/energy transfer processes. Very recently, a terpyridine (tpy) derivative, as a ligand for Fe(II) ions, was used to modify graphene oxide [51]. More precisely, Fe(II) coordinates tpy groups of the upper surface of one of the graphene sheets with the same groups of the lower surface of another graphene sheet, promoting the self-assembly of GO. The Fe–tpy–GO nanohybrid fully decomplexes when treated with EDTA and re-complexes after fresh addition of Fe(II) (Scheme 2). Furthermore, Fe–tpy–GO was tested as a catalyst for the oxygen reduction reaction (ORR) and found to be durable against carbon monoxide poisoning and to exhibit a higher fuel selectivity compared with commercially available Pt/C electro-catalysts. The ORR reactivity is tightly connected to electron transfer processes, thus indicating indirectly the occurrence of such phenomena. Ruthenium(II) was also employed as a coordinating cation and resulted in a Ru–tpy–GO hybrid material with an enhanced photocurrent response, higher than GO and Fe–tpy–GO, probably due to a more effective electron transfer from the Ru–tpy core to the graphene network. This class of self-assembled covalently functionalized GO-based nanohybrids is a promising new member in the family of graphene hybrid materials for potential applications in energy conversion and storage.

**Scheme 3:**
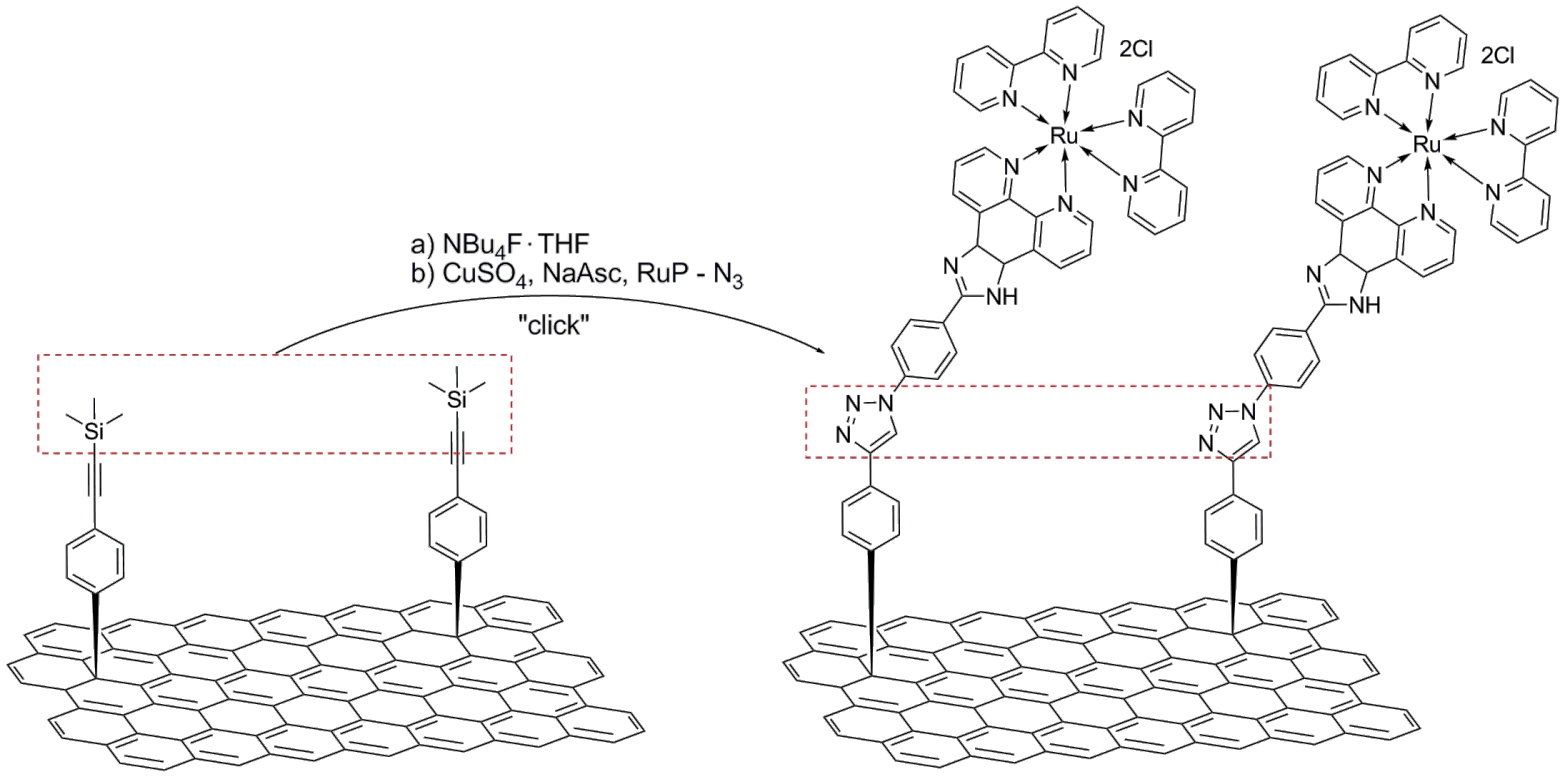
“Click” reaction for the grafting of a porphyrin onto reduced graphene oxide sheets that was pre-modified by phenylacetylene units [52].

On similar grounds, rGO was initially modified by phenylacetylene units, which were used to mediate the grafting of zinc–porphyrin (ZnP) and ruthenium–phenanthroline (RuP) chromophores through a copper-catalyzed “click” chemistry reaction [52]. The preparation of the RuP–rGO hybrid material is illustrated in Scheme 3. The excellent dispersibility of the two novel types of graphene-based nanostructures in common organic solvents allowed for the easy characterization by various microscopy and spectroscopic techniques. Spectroscopic results of the ground and excited states gave important insights about the electronic communication between the chromophore moiety and rGO, which lead to the fabrication of photoelectrochemical cells. The photo-induced electron-transfer properties of ZnP–rGO and RuP–rGO were evaluated by examining the photocurrent responses in thin films on indium tin oxide (ITO).

In all the above studies, the hybrid materials were applied for energy conversion. However, the unique properties of graphene and in particular its ability to efficiently quench the photo-induced emission of electron donors have recently been used for other purposes. Thus, it is interesting to note that, although graphene-based biomaterials are out of the scope of this mini-review, GO has been covalently functionalized with peptides, antibodies and other biomolecules for applications in diagnostics, novel therapeutic approaches and near infrared (NIR) photo-thermal therapies [53]. A representative work demonstrates the usage of a GO–coumarin conjugate as an activated fluorescent imaging probe with high sensitivity in the visualization of cancer cells [54]. The fluorescence of the probe can be switched off or on during intracellular imaging. Normally, the probe shows no or weak fluorescence (off) due to the fluorescence quenching of the coumarin moiety. However, fluorescence is activated (on) and significantly enhanced inside the cells by glutathione-initiated dissociation.

### Donor–acceptor hybrids based on exfoliated graphene

**Figure 4:**
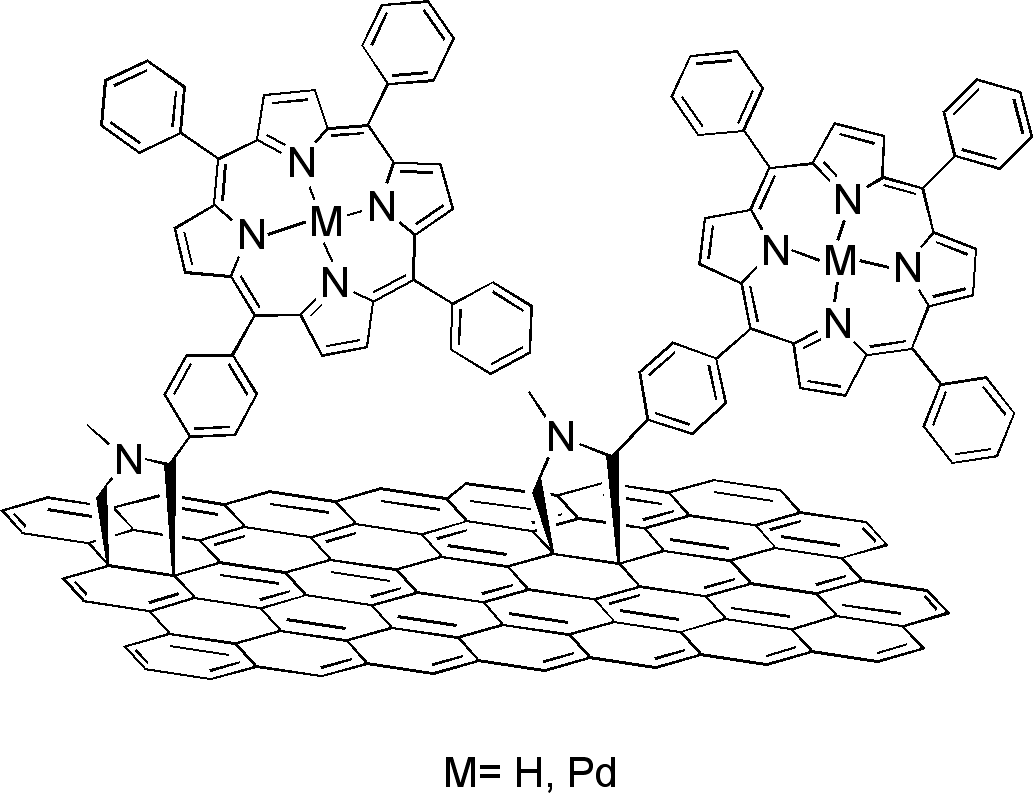
Free and Pd-metallated tetraphenylporphyrin moieties as substituents of pyrrolidine rings covalently anchored to exfoliated graphene [55].

However, as mentioned earlier, the surface defects in the sp^2^-network of GO influence the electronic and optical properties of the hybrid materials. Therefore, free TPP as well as Pd-metallated TPP were added to exfoliated graphene trough a 1,3-dipolar cycloaddition of azomethine ylides [55]. In this approach, graphite flakes were sonicated in *o*-DCB to induce exfoliation. In the following step, sarcosine and the corresponding aldehydes bearing the TPP and Pd–TPP moieties were added and the reaction mixture was stirred at 160 °C under N_2_ for one week to yield the structures shown in Figure 4. The presence of TPP or Pd–TPP in the graphene-based hybrid materials was confirmed by standard spectroscopic methods, such as UV–vis, Raman, FTIR and XPS. Complementary electron microscopy studies showed that the functionalization processes did not affect the morphology of graphene in the hybrid materials. More importantly, fluorescence and phosphorescence quenching of either the free or the Pd-metallated porphyrin was observed, with a simultaneous decrease on the fluorescence emission lifetimes. These observations are indicative of intrahybrid interactions in the excited state through energy- and/or electron-transfer between graphene and the covalently bound porphyrin moieties. It is noteworthy, that the particular type of functionalization resulted in a relatively low loading of porphyrins on graphene, as calculated by thermogravimetry. Thus, the π-electronic network of graphene was preserved to large extent, which renders the hybrid materials suitable for applications in solar and/or photoelectrochemical cells.

Another graphene-based hybrid material was prepared in which a porphyrin was anchored to graphene through Suzuki coupling of iodophenyl-functionalized graphene and the corresponding porphyrin boronic ester [56]. The covalently linked graphene–porphyrin hybrid was especially designed to possess a short, yet rigid phenylene spacer between porphyrin and graphene. Photophysical measurements revealed the presence of a short-lived porphyrin singlet excited state (38 ps), however, without yielding the porphyrin radical cation, thus suggesting that energy transfer from the porphyrin excited state to the graphene sheets occurs. Moving a step forward, a prototype photoelectrochemical device, in which a SnO_2_ electrode was coated with the aforementioned graphene–porphyrin hybrid material, was constructed. Photophysical and electrical measurements revealed the absence of any photocurrent response from the porphyrin absorption, leading to a maximum IPCE value of approximately 3%.

**Figure 5:**
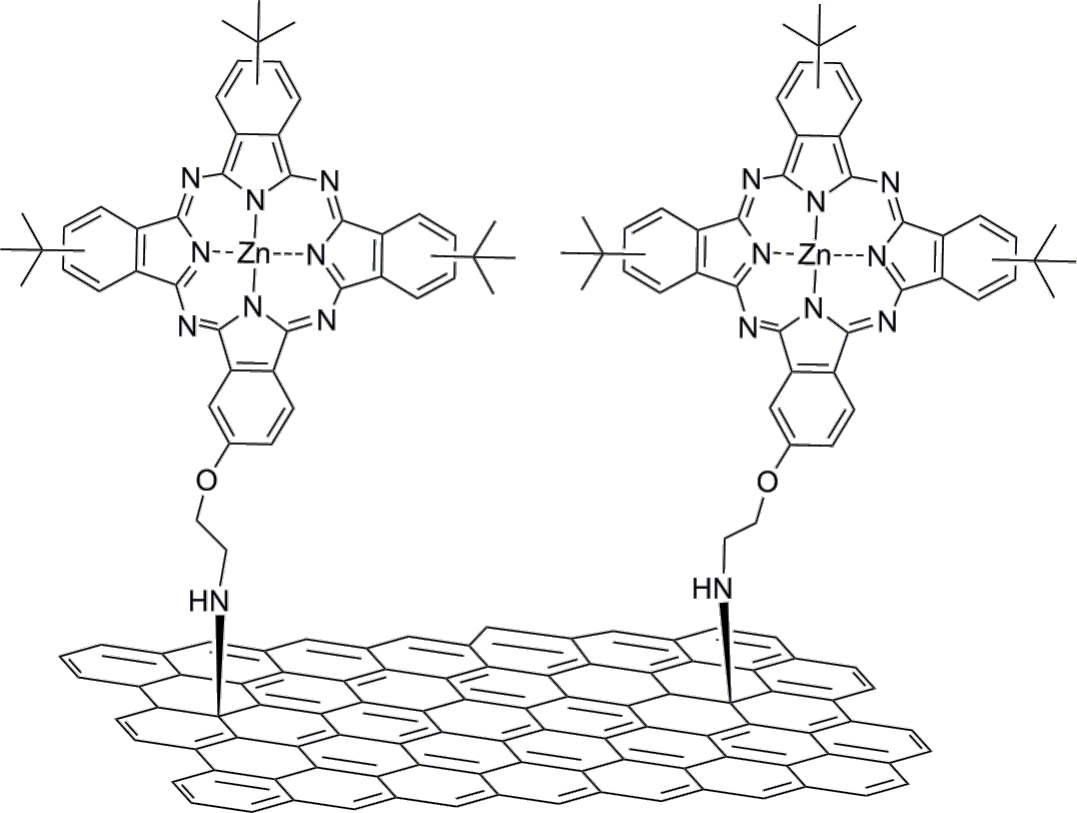
Covalent grafting of (2-aminoethoxy)(tri-*tert*-butyl)phthalocyanine zinc to exfoliated graphene sheets via direct nucleophilic addition of primary amines [57].

A further survey of the functionalization of graphene with organic electron donors revealed that phthalocyanines were introduced on exfoliated graphene in parallel and via two independent routes. In the first approach, the covalent grafting of (2-aminoethoxy)(tri-*tert*-butyl)phthalocyanine zinc (ZnPc) to exfoliated graphene sheets via direct nucleophilic addition of primary amines was accomplished (Figure 5) [57]. The ZnPc–graphene hybrid material was extensively characterized by complementary spectroscopic means as well as electron microscopy. Efficient fluorescence quenching of ZnPc in the ZnPc–graphene hybrid material was observed and the deactivation pathway was evaluated by using femtosecond transient absorption spectroscopy. The charge-separated state ZnPc^•+^–graphene^•−^ was identified. An electrode of the ZnPc–graphene hybrid material was prepared and its photoelectrochemical properties were examined. It was found to exhibit stable and reproducible photocurrent responses, and the incident-photon-to-current conversion efficiency was determined to be 2.2% at 420 nm. These results highlight the important role of the covalent grafting of ZnPc onto the graphene sheet for enhancing the photo-induced electron-transfer phenomena and achieving higher IPCE values as compared to non-covalently interacting ZnPc [58], or ZnPc-functionalized (phenylene vinylene) oligomers [59].

**Figure 6:**
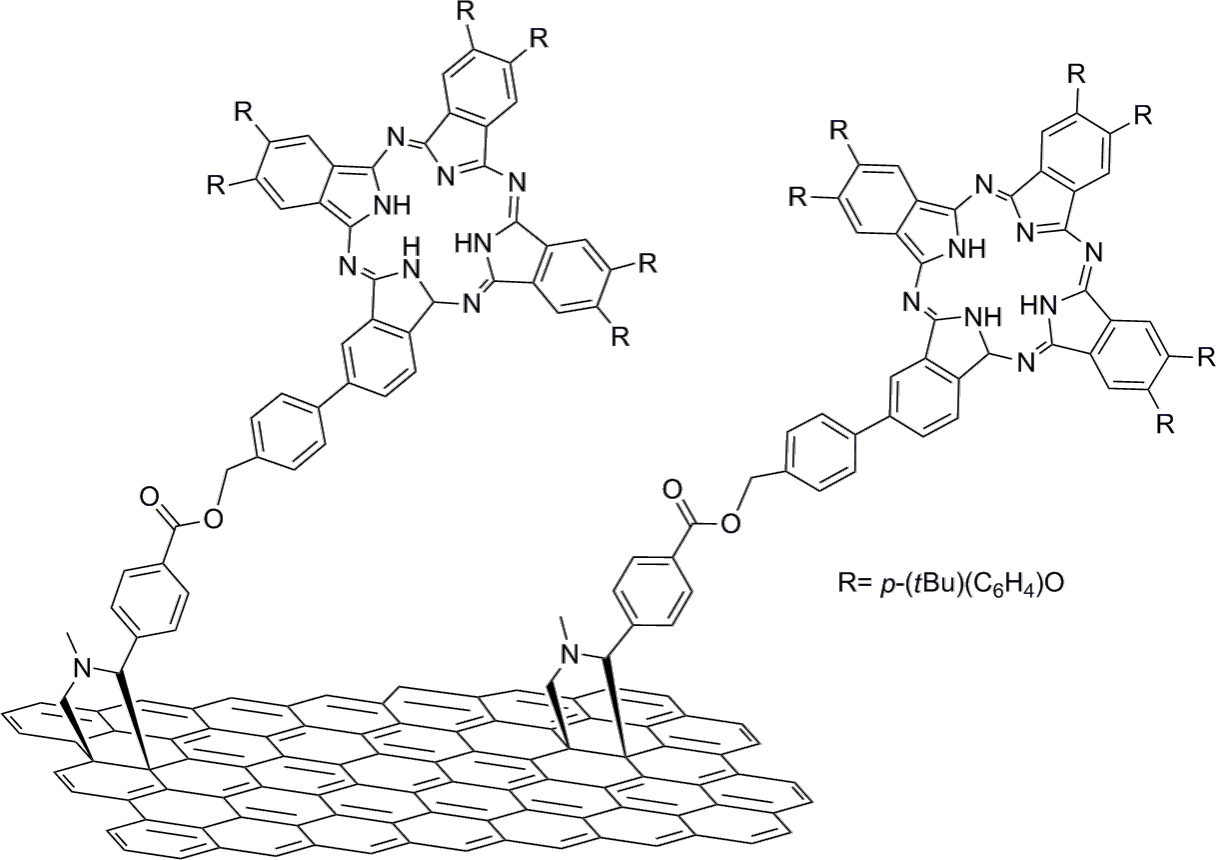
Phthalocyanine–graphene hybrid material, prepared upon condensation of mono-OH-derivatized phthalocyanine with pendant phenylcarboxylic acid units present as substituents of pyrrolidine rings on pre-modified graphene sheets [60].

In the second strategy followed for the realization of a Pc–graphene hybrid, few-layered graphene, obtained by exfoliating graphite through sonication, was utilized. The exfoliated graphene sheets were then reacted with excess *N*-methylglycine and 4-formylbenzoic acid to yield functionalized graphene that carries pyrrolidine rings with pendant phenylcarboxylic acid units. In the next step, the condensation between the –COOH units of the pre-functionalized graphene and mono-OH-derivatized phthalocyanine, in the presence of EDC/HOBt as activators, resulted in the graphene–Pc hybrid material (Figure 6) [60]. Photoexcitation of Pc–graphene and evaluation of the species by pump-probe femtosecond transient absorption spectroscopy revealed the formation of the radical anion of graphene through a broad band in the NIR region. Based on multi-wavelength analysis, a short-lived and a long-lived component with lifetimes of 3.3 and 270 ps, respectively, in DMF were identified, suggesting that the ultra fast charge-separation in the Pc–graphene is followed by a slower charge-recombination.

**Figure 7:**
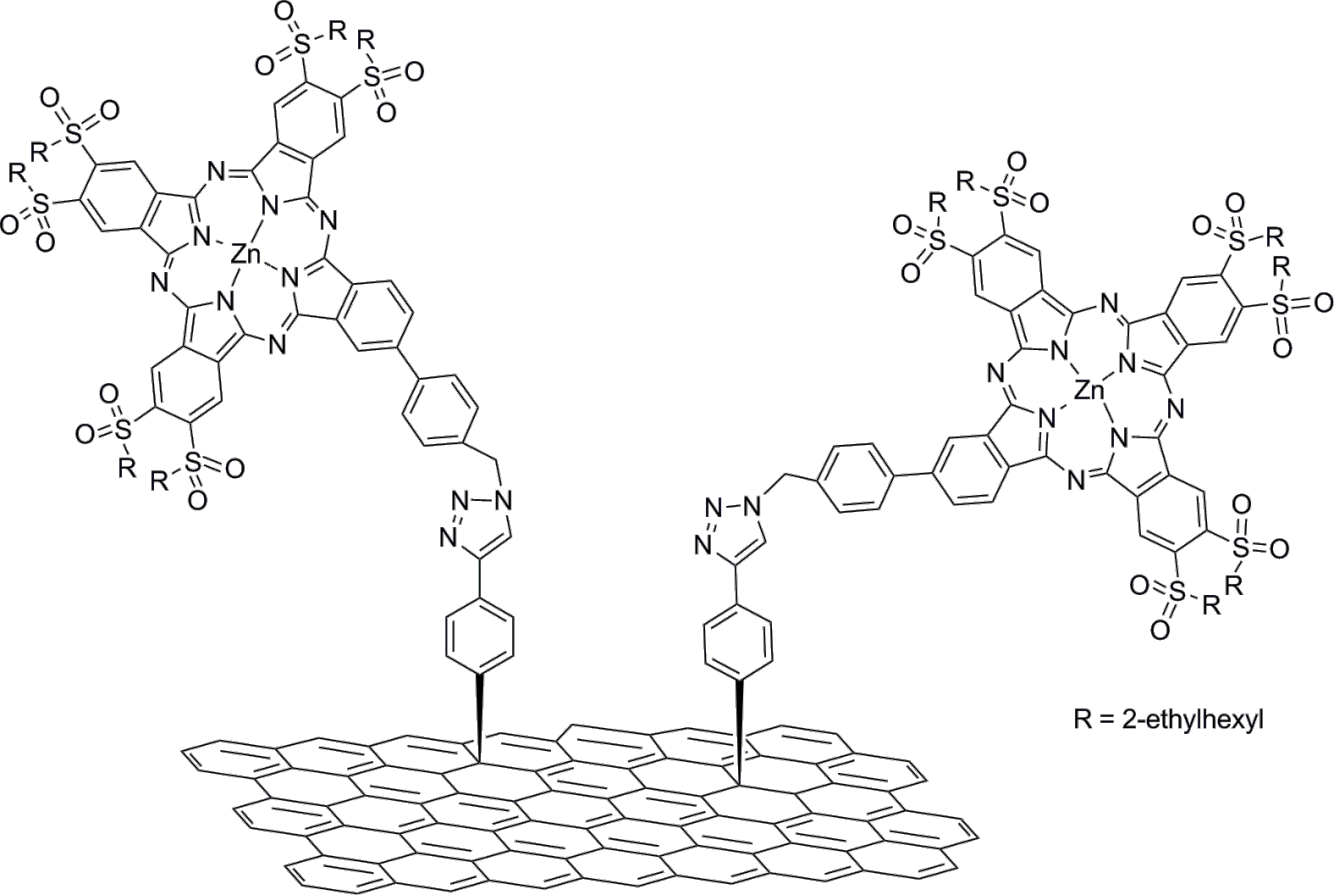
Sulfonyl-substituted zinc phthalocyanine covalently bound to pre-modified graphene via “click” chemistry [61].

Recently, a sulfonyl-substituted zinc phthalocyanine was synthesized and covalently bound to exfoliated graphene via “click” chemistry (Figure 7). Interestingly, due to the strong electron-withdrawing effect of the sulfonyl units, the particular phthalocyanine component acts as electron acceptor in the graphene–ZnPc hybrid material [61]. Complementary electrochemical measurements and photophysical assays, based on fluorescence emission and transient absorption spectroscopy, verified that electron transfer occurs from graphene to ZnPc.

**Figure 8:**
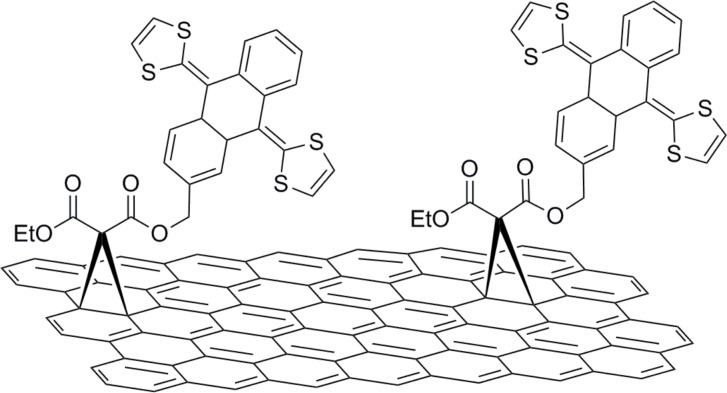
Extended tetrathiafulvalene units covalently attached to exfoliated graphene via Bingel cycloaddition reaction [39].

Beyond the coupling of porphyrin and phthalocyanine dyes to graphene, other photo- and/or electro-active units have been also introduced. In this context, an extended tetrathiafulvalene (exTTF) was grafted to exfoliated graphene via a microwave-assisted Bingel reaction (Figure 8) [39]. The new exTTF-graphene material was fully characterized by spectroscopic, thermal, and microscopy means. Importantly, the electrochemical properties of exTTF-graphene were studied by cyclic voltammetry which allowed identification of the formation of a radical ion pair that includes one-electron oxidation of exTTF and one electron reduction of graphene.

**Figure 9:**
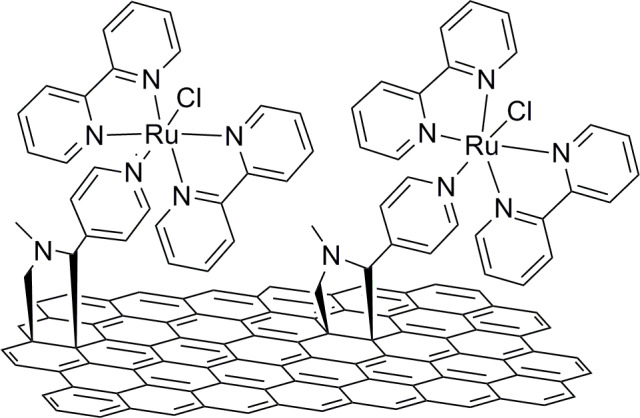
Graphene sheets covalently functionalized with a Ru-bipyridine complex [62].

Covalently functionalized few-layer graphene with the bipyridine ruthenium complex (2,2′-bipyridyl)-4-pyridyl-ruthenium(II)chloride, Ru(bpy)_2_(py)Cl, results in the formation of another novel nanohybrid material (Figure 9). A slow recombination of the photoexcited charges was identified and demonstrates the high potential of the hybrid material for immediate applications in photocatalysis as well as in solar energy conversion schemes [62]. The Ru(bpy)_2_(py)Cl–graphene hybrid material highlights the importance of the covalent binding and the chemistry chosen for the particular functionalization methodology for the enhancement of photo-induced electron transfer. It is noteworthy that triazole rings constructed by “click” reaction of an alkyne-functionalized graphene sheet (or chromophore) and an azide-functionalized chromophore (or graphene sheet) were also shown to amplify the charge transfer [52].

An overview of common photophysical properties for some of the discussed graphene-based nanohybrids is given in Table 1.

**Table 1:** Some photophysical properties for selected graphene-based hybrid materials.

hybrid material	references	IPCE^a^	τ_CS_ / ps^b^	*k*^CS^ / s^−1 c^	Φ^CS d^	τ_CR_ / ns^e^	*K*^CR^ / s^−1 f^	Δ*G*_CS_ / eV^g^

GO–TPP	Figure 2 and [44]	1.3%	675	1.14 × 10^9^	0.77	56	1.8 × 10^7^	−1
exfoliated graphene–TPP	Figure 4 and [55]	—	<500	—	0.3	—	—	—
exfoliated graphene–Pd-TPP	Figure 4 and [55]	—	8,000	—	<0.01	—	—	—
exfoliated graphene–ZnPc	Figure 5 and [57]	2.2%	7.7	—	0.98	4.3	2.3 × 10^8^	—
exfoliated graphene–Pc	Figure 6 and [60]	—	3.3	2.03 × 10^9^	0.7	—	—	—
exfoliated graphene–ZnPc	Figure 7 and [61]	—	1	—	—	—	—	—
exfoliated graphene–exTTF	Figure 8 and [39]	—	—	—	—	—	—	0.69
exfoliated graphene–TPP	[31]	3%	30	—	—	—	—	—

^a^The incident-photon-to-current conversion efficiency of the fabricated devices. ^b^Charge-separation lifetime of the photo-excited hybrids. ^c^Charge-separation rate constant. ^d^Quantum yield for charge-seperation. ^e^Charge-recombination lifetime. ^f^Charge-recombination rate constant. ^g^Free energy of the charge-seperated state.

## Conclusion

To summarize, recent advances in the synthesis of graphene sheets that are covalently functionalized with photo- and/or electro-active moieties for the formation of novel hybrid materials were reviewed. Although in early stages oxidation was the method of choice for the functionalizing graphene, the introduction of numerous lattice defects that significantly alter the electronic properties of graphene, required the development of new routes for obtaining graphene sheets that preserve to a large extent the high conductivity of the material. Therefore, graphene-based hybrid materials are formed by exfoliating graphite and a subsequent chemical functionalization. Such graphene-based donor–acceptor hybrid materials were found to possess interesting optical and photophysical properties. Charge-transfer phenomena mainly from the photoexcited organic chromophore units to the graphene layers were observed. Although the potential of these materials to be applied in solar and photoelectrochemical cells is great, there is surely a need for the preparation and the evaluation of more graphene-based materials in order to fully understand and describe the electronic interaction processes of these systems.

## Acknowledgements

Partial financial support from GSRT/NSRF 2007-2013 through action “ARISTEIA II” project FUNGRAPH (3150) “Functionalization of graphene with multichromophoric arrays of photoactive units for energy conversion” is acknowledged.
